# Beyond conventional biomarkers: the role of alpha-fetoprotein in gastroenteropancreatic neuroendocrine neoplasms

**DOI:** 10.3389/fendo.2026.1778029

**Published:** 2026-03-19

**Authors:** Anna La Salvia, Giuseppe Fanciulli

**Affiliations:** 1 National Center for Drug Research and Evaluation, National Institute of Health (ISS), Rome, Italy; 2 Neuroendocrine Tumor Unit, Department of Medicine, Surgery and Pharmacy, University of Sassari, Sassari, Italy; 3 Endocrine Unit, Azienda Ospedaliero-Universitaria (AOU) of Sassari, Sassari, Italy

**Keywords:** AFP, circulating biomarkers, disease progression, gastroenteropancreatic neuroendocrine neoplasms, prognosis, tumor grade

## Abstract

**Background:**

Gastroenteropancreatic neuroendocrine neoplasms (GEP-NENs) represent a diverse group of tumors, ranging from well-differentiated neuroendocrine tumors to poorly differentiated neuroendocrine carcinomas. Current biomarkers for GEP-NENs are weak and lack sufficient sensitivity and specificity, complicating diagnosis and prognosis. Alpha-fetoprotein (AFP), a well-established biomarker in other cancers, has been reported as a potentially useful biomarker also for GEP-NENs, but its clinical relevance remains unclear. This narrative review evaluates AFP’s role in GEP-NENs.

**Materials and methods:**

We conducted a systematic search of PubMed, Scopus, and Web of Science up to December 1, 2025, using MeSH terms and free-text keywords related to AFP and GEP NENs. Only studies reporting measurable circulating AFP levels in GEP-NENs patients were included. Eligible study designs comprised retrospective or prospective studies and interventional trials. Non-English publications, case reports, small case series, reviews, and editorials were excluded.

**Results:**

We identified ten studies evaluating circulating AFP in GEP-NENs, comprising nine retrospective cohorts’ studies and one prospective study. 2,132 patients were included, with AFP measurements available for 1,222. AFP elevation was rare in GEP-NENs but consistently associated with advanced disease, high tumor grade, and increased proliferative activity. Elevated AFP levels correlated with poorer survival outcomes and higher treatment response rates in patients undergoing chemotherapy. However, despite these associations, the prognostic value of AFP diminished after adjusting for clinicopathological factors, limiting its role as an independent biomarker.

**Conclusions:**

Current evidence suggests that AFP identifies biologically aggressive subsets of GEP-NENs, reflecting disease burden in specific contexts. While AFP should not be considered an independent biomarker, it holds potential as a contextual signal of aggressive tumor biology and as an adjunctive tool within integrated clinical and pathological frameworks.

## Graphical Abstract

**Figure:**
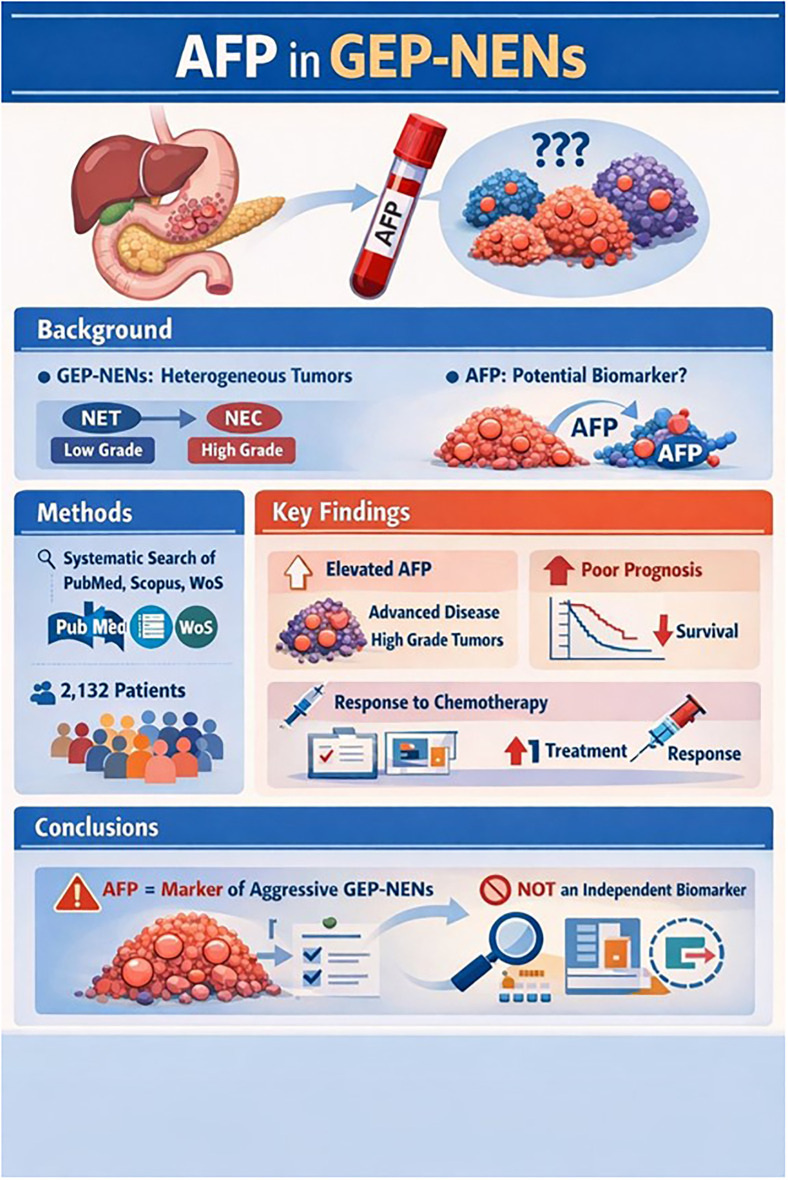
Our review of circulating AFP in GEP-NENs highlights its association with tumor aggressiveness and potential role as an adjunctive biomarker.

## Introduction

Gastroenteropancreatic neuroendocrine neoplasms (GEP-NENs) represent a biologically and clinically heterogeneous group of malignancies originating from neuroendocrine cells distributed throughout the gastrointestinal tract and pancreas. Their clinical behavior encompasses a wide spectrum, ranging from well-differentiated neuroendocrine tumors (NETs), often characterized by indolent growth and prolonged survival, to poorly differentiated neuroendocrine carcinomas (NECs), which typically display aggressive clinical courses and limited therapeutic options (1). This heterogeneity poses significant challenges in diagnosis, prognostic stratification, and treatment selection.

Current pathological classification systems reflect this complexity. The contemporary WHO framework categorizes GEP-NENs according to both morphological differentiation and proliferative activity, as assessed by mitotic count and Ki-67 index, distinguishing between NETs graded G1 to G3 and NECs as a distinct entity (2). This taxonomy, developed through international expert consensus, has substantially improved standardization across clinical practice and research. Despite this progress, it has also become increasingly evident that tumors sharing similar grade and stage may exhibit markedly divergent clinical trajectories, suggesting that conventional histopathological parameters alone do not fully capture the underlying biological diversity of GEP-NENs.

While histopathological classification provides valuable insights into GEP-NENs, molecular profiling has emerged as a complementary tool that offers deeper insights into the tumor’s biological basis. Genomic and molecular studies have demonstrated that well-differentiated NETs and NECs are characterized by distinct molecular landscapes, supporting the concept that differentiation status reflects fundamental biological differences rather than a simple continuum of proliferative activity (3, 4). Nevertheless, the integration of molecular data into routine clinical decision-making remains limited, reinforcing the continued reliance on clinical, pathological, and biochemical markers.

The clinical management of GEP-NENs is therefore inherently multidisciplinary, combining histopathological evaluation, anatomical and functional imaging, and the measurement of circulating biomarkers tailored to tumor subtype and secretory phenotype (5). Major international guidelines emphasize the role of biochemical markers for diagnosis, prognostication, and disease monitoring in neuroendocrine neoplasms (NENs) (5–7). However, despite their widespread use, currently available circulating biomarkers suffer from well-recognized limitations. These include variable sensitivity and specificity, assay heterogeneity, susceptibility to nontumor-related confounders, and inconsistent correlation with tumor burden or clinical outcomes, all of which complicate their routine application in clinical practice (8).

These shortcomings have fueled interest in identifying additional circulating biomarkers that may better reflect biologically relevant tumor features, such as differentiation status, proliferative activity, or aggressive behavior, and that could improve risk stratification or therapeutic decision-making. Alpha-fetoprotein (AFP) is best known as a serum biomarker for hepatocellular carcinoma and germ cell tumors, where it has established diagnostic and surveillance roles (9, 10). Beyond these settings, AFP elevation has been reported in tumors exhibiting hepatoid differentiation, a rare morphological and functional phenotype in which extrahepatic carcinoma cells acquire hepatocellular-like features and the capacity to produce AFP (11).

Importantly, these observations suggest that AFP expression is more closely linked to lineage differentiation and cellular plasticity than to organ specificity per se. From a biological standpoint, AFP production may therefore represent a surrogate of aberrant differentiation programs or dedifferentiation processes, phenomena that have been associated with aggressive tumor behavior across multiple malignancies. Within GEP-NENs, this raises the hypothesis that AFP elevation might identify biologically distinct subsets characterized by higher proliferative activity, advanced disease stage, or unfavorable clinical outcomes.

To date, evidence regarding the role of AFP in GEP-NENs remains fragmented across studies. Individual analytical studies have reported associations between elevated circulating AFP levels and advanced stage, high tumor grade, increased tumor burden, or reduced survival, while others have failed to demonstrate independent prognostic or diagnostic value once established clinicopathological variables are considered. The interpretation of these findings is further complicated by heterogeneity in study design, AFP cut-off definitions, patient populations, and outcome measures, as well as by the retrospective nature of most available data.

Moreover, the evaluation of AFP in GEP-NENs exemplifies broader challenges inherent to prognostic biomarker research, including the risk of overinterpreting univariate associations, inadequate control for confounding variables, and variability in analytical methodologies. These issues underscore the need for careful synthesis and critical appraisal of existing evidence before proposing the integration of additional biomarkers into clinical practice.

To the best of our knowledge, no comprehensive narrative review has systematically examined the available analytical studies assessing circulating AFP in patients with GEP-NENs, integrating biological rationale with clinical and methodological considerations. Addressing this gap is particularly relevant given the ongoing need for improved biomarkers capable of refining patient stratification and complementing existing diagnostic and prognostic tools.

Therefore, the aim of this narrative review is to critically evaluate the current evidence on circulating AFP in GEP-NENs, focusing on its associations with histopathological subtypes, tumor burden, metastatic patterns, and clinically relevant outcomes such as progression-free and overall survival. By synthesizing available data and highlighting methodological limitations, this review seeks to clarify whether AFP represents a meaningful biomarker signal in selected clinical contexts or whether its observed associations primarily reflect broader features of tumor aggressiveness rather than independent clinical utility.

## Materials and methods

We searched three bibliographic databases (PubMed, Scopus, and Web of Science) from inception to December 1^st^, 2025. The search strategy combined Medical Subject Headings (MeSH) and free-text terms: (“alpha-fetoprotein” OR “AFP”) AND (“neuroendocrine tumor” OR “neuroendocrine carcinoma” OR carcinoid OR “gastrointestinal neuroendocrine tumor” OR “pancreatic neuroendocrine tumor”). Non-English articles were excluded,

We included only analytical human studies, defined as original research designed to evaluate associations between exposures/interventions and outcomes, including retrospective or prospective cohort studies, case-control studies, and interventional trials. Only studies reporting measurable circulating AFP levels in patients with confirmed GEP-NENs (NETs, NECs, or MiNENs) were included. Case reports, case series, reviews and editorials were excluded.

Given the temporal evolution of the WHO classification of NENs, including changes in nomenclature and grading, we retained the original terminology adopted in each study. Reclassification of historical cohorts according to the 2022 WHO criteria could introduce interpretative bias due to differences in diagnostic definitions; therefore, original terminology was preserved to ensure contextual accuracy (12).

Case reports, small case series, reviews, and editorials were excluded due to their limited generalizability, high susceptibility to bias, absence of control groups, and inability to support robust inference on biomarker performance, which could lead to misleading conclusions if extrapolated to broader clinical populations (13, 14).

## Results

We identified ten studies evaluating circulating AFP in GEP-NENs, comprising nine retrospective cohorts’ studies and one prospective study. 2,132 patients were included, with AFP measurements available for 1,222. Study data are summarized in **Tables 1a**-**1c**.

**Table 1a:** Study design and patient demographics (Global population).

Year	Author	Study design	Disease cohort	Total N	Patient age (Years)	Sex (M/F)	Disease stage	Tumor grade
2008	Shah et al. (15)	Clinical database analysis	Mixed NENs (GEP-NETs unspecified)	360	Not provided	Not provided	Not specified	G1 (≤2%), G2 (3-20%), G3 (≥20%)
2010	Turner et al. (16)	Retrospective chemotherapy cohort	Advanced NENs (60% pancreatic)	82	Median: 55.4 (range 22.2–81.6)	42/40	89% metastatic disease	G1: 8, G2: 47, G3: 15
2019	Li et al. (17)	Retrospective comparative study	PHNENs vs PanNENs	44	PHNENs: 55.9 ± 13.0, PanNENs: 56.7 ± 11.2	PHNENs: 7/5, PanNENs: 16/16	Not reported	PHNENs: 7 NEC, 5 NET G2, PanNENs: 11 NEC, 21 NET G1/G2
2019	Zhou et al. (18)	Retrospective model development	PanNETs	91	G1: 52.47 ± 11.70, G2: 49.19 ± 11.34, G3: 50.00 ± 17.04	G1: 14/18, G2: 37/11, G3: 4/7	Not reported	G1: 32, G2: 48, G3: 11
2021	Li et al. (19)	Retrospective nomogram development	Healthy controls, patients with benign diseases, patients with cancers and GEP-NETs	1,014	Mean: 54.36 ± 12.64, Median: 56.0 (range 17–81)	106/105	Not reported	G1, G2, G3 according to WHO classification
2022	Yu et al. (20)	Retrospective diagnostic study	Non-metastatic PanNENs	142	Ki-67 ≤5%: Median 48, Ki-67 >5%: Median 57	Ki-67 ≤5%: 57/47, Ki-67 >5%: 21/17	Non-metastatic	Ki-67 ≤5%, Ki-67 >5%
2023	Rosiek et al. (21)	Observational cohort study	PanNENs	115	Mean: 53, Median (BMets 57, No BMets 55)	49/66	Stage I–IV (38% stage IV)	G1: 45%, G2: 39%, G3: 3%, NEC: 4%
2025	Jumai et al. (22)	Retrospective model development	Well-differentiated GEP-NENs	200	Median: 51.4 (IQR: 40–59)	103/97	Stratified by LTB	G1: 21%, G2: 78%, G3: 1%
2025	Musiałkiewicz et al. (23)	Longitudinal observational study	Liver-metastatic PanNETs	41	Mean: 63.1 ± 9.2	12/29	All patients had liver metastasis	G1: 46.3%, G2: 53.7%
2025	Wan et al. (24)	Single-center retrospective study	PanNETs	43	Median: 58 (range 26–80), Mean: 58.4 ± 2.8	20/23	AJCC stages I–IV	G1: 28.0%, G2: 58.1%, G3: 13.9%

AFP, Alpha-fetoprotein; AJCC, American Joint Committee on Cancer; BMets, Bone Metastases; G, Grade; GEP-NENs, Gastroenteropancreatic Neuroendocrine Neoplasms; GEP-NETs, Gastroenteropancreatic Neuroendocrine Tumors; IQR, Interquartile Range; LTB, Liver Tumor Burden; NEC, Neuroendocrine Carcinoma; NEN, Neuroendocrine Neoplasm; NET, Neuroendocrine Tumor; PanNEN, Pancreatic Neuroendocrine Neoplasm; PanNET, Pancreatic Neuroendocrine Tumor; PHNEN, Primary Hepatic Neuroendocrine Neoplasm.

**Table 1b:** AFP-Specific subgroup demographics (Population on whom AFP was measured).

Year	Author	AFP subgroup Size	Patient age (Years) (AFP subgroup)	Sex (M/F) (AFP subgroup)	Disease stage (AFP subgroup)	Tumor grade (AFP subgroup)
2008	Shah et al. (15)	294	Mean: Elevated AFP 51, Controls 48	Elevated AFP: 15/13, Controls: 18/22	Elevated AFP: 24/28 (86%) stage IV, Controls: 25/40 (63%)	G1 (≤2%), G2 (3-20%), G3 (≥20%)
2010	Turner et al. (16)	57	Not separated	Not separated	Metastatic: 76% with liver metastasis	G1: 8, G2: 47, G3: 15
2019	Li et al. (17)	44	PHNENs: 55.9 ± 13.0, PanNENs: 56.7 ± 11.2	PHNENs: 7/5, PanNENs: 16/16	Not specified	PHNENs: 7 NEC, 5 NET G2, PanNENs: 11 NEC, 21 NET G1/G2
2019	Zhou et al. (18)	91	G1: 52.47 ± 11.70, G2: 49.19 ± 11.34, G3: 50.00 ± 17.04	G1: 14/18, G2: 37/11, G3: 4/7	Not specified	G1: 32, G2: 48, G3: 11
2021	Li et al. (19)	211	Mean: 54.36 ± 12.64, Median: 56.0 (range 17–81)	106/105	Not specified	G1, G2, G3 according to WHO classification
2022	Yu et al. (20)	126	Ki-67 ≤5%: Median 48, Ki-67 >5%: Median 57	Ki-67 ≤5%: 57/47, Ki-67 >5%: 21/17	Non-metastatic	Ki-67 ≤5%, Ki-67 >5%
2023	Rosiek et al. (21)	115	Mean: 53, Median (BMets 57, No BMets 55)	49/66	Stage I–IV (38% stage IV)	G1: 45%, G2: 39%, G3: 3%, NEC: 4%
2025	Jumai et al. (22)	200	Median: 51.4 (IQR: 40–59)	103/97	Stratified by LTB	G1: 21%, G2: 78%, G3: 1%
2025	Musiałkiewicz et al. (23)	41	Mean: 63.1 ± 9.2	12/29	All patients had liver metastasis	G1: 46.3%, G2: 53.7%
2025	Wan et al. (24)	43	Median: 58 (range 26–80), Mean: 58.4 ± 2.8	20/23	AJCC stages I–IV	G1: 28.0%, G2: 58.1%, G3: 13.9%

AFP, Alpha-fetoprotein; AJCC, American Joint Committee on Cancer; BMets, Bone Metastases; G, Grade; IQR, Interquartile Range; LTB, Liver Tumor Burden; NEC, Neuroendocrine Carcinoma; NET, Neuroendocrine Tumor; PanNEN, Pancreatic Neuroendocrine Neoplasm; PHNEN, Primary Hepatic Neuroendocrine Neoplasm.

**Table 1c:** AFP-Specific findings (AFP-Related findings in subgroup).

Year	Author	AFP cutoff/value	AFP-specific objective	Primary AFP result	Key AFP findings
2008	Shah et al. (15)	≥1.5 × ULN; Mean: 273.8 ng/mL	Diagnostic and prognostic assessment	Elevated AFP in 9.5%	Elevated AFP associated with advanced stage and shorter survival (37.6 vs 69.0 months; P = 0.001); independent association with survival on multivariable analysis
2010	Turner et al. (16)	>16 ng/mL (1.5 × ULN)	Prognostic and predictive factors	Higher response rate to chemotherapy	AFP >16 ng/mL associated with higher chemotherapy response; not prognostic for OS on multivariable analysis
2019	Li et al. (17)	>20 ng/mL; Median: 2.6 vs 3.0 ng/mL	Prognostic evaluation	Prognostic on univariate analysis	AFP >20 ng/mL associated with worse OS on univariate analysis; not independent on multivariate analysis
2019	Zhou et al. (18)	Mean: G1 2.27, G2 3.34, G3 3.47 ng/mL	Grade prediction using ML	AFP correlated with grade	Higher AFP in G2 and G3 vs G1; AFP included as ML feature
2021	Li et al. (19)	Mean: Total 12.01, G1 2.55, G2/3 25.64 ng/mL	Preoperative grade prediction	Univariate association	AFP associated with grade on univariate analysis; excluded from final nomogram
2022	Yu et al. (20)	Mean: 4.02 vs 19.20 ng/mL; AUC 0.626	Predict Ki-67 index	AFP higher in high Ki-67 group	AFP significantly associated with Ki-67 >5% (P = 0.030)
2023	Rosiek et al. (21)	Median 3 µg/L	Detect BMets	No discriminatory value	AFP did not differ between patients with and without BMets
2025	Jumai et al. (22)	AFP/ULN ratio: 0.13 vs 0.16	Predict LTB	Higher AFP ratio in high LTB group	AFP ratio significant on univariate analysis; excluded from final LASSO model
2025	Musiałkiewicz et al. (23)	Serial AFP (IU/mL)	Longitudinal AFP dynamics	AFP increased over time	Rising AFP associated with higher grade, greater LTB, and disease progression
2025	Wan et al. (24)	AUC 0.658; cutoff 15.5 ng/mL	Diagnostic efficacy	Fair diagnostic accuracy	AFP positivity rare across stages and grades

AFP, Alpha-fetoprotein; ALT, Alanine Aminotransferase; AUC, Area Under the Curve; BMets, Bone Metastases; CA19-9, Carbohydrate Antigen 19-9; CA125, Carbohydrate Antigen 125; CEA, Carcinoembryonic Antigen; CI, Confidence Interval; GGT, Gamma-Glutamyl Transferase; HR, Hazard Ratio; LDH, Lactate Dehydrogenase; LASSO, Least Absolute Shrinkage and Selection Operator; LTB, Liver Tumor Burden; ML, Machine Learning; NSE, Neuron-Specific Enolase; OS, Overall Survival; ROC, Receiver Operating Characteristic; ULN, Upper Limit of Normal.

Early evidence linking circulating AFP to aggressive disease biology in NETs emerged from a large retrospective clinical database analysis by Shah et al. (2008) (15). The study included a total of 360 patients, with 294 patients having available AFP measurements. Among these patients, 28 (9.5%) had elevated AFP levels, defined as ≥1.5 times the upper normal limit. The mean age of the AFP-elevated group was 51 years, with a gender distribution of 15 males and 13 females, while the control group had a mean age of 48 years and a gender distribution of 18 males and 22 females. Patients with elevated AFP levels were significantly more likely to present with advanced disease, with 86% of the elevated AFP group having stage IV tumors, compared to 63% in the control group. The mean survival for patients with elevated AFP was 37.6 months, which was significantly shorter than the 69.0 months observed for patients with normal AFP levels (P = 0.001). AFP levels showed positive correlations with other biomarkers, including human chorionic gonadotrophin-*β* (hCGβ) (r = 0.558), chromogranin A (r = 0.451), and Ki-67 index (r = 0.381, P = 0.007). In contrast, AFP levels were inversely correlated with overall survival (OS) (r = -0.419, P = 0.001). Notably, AFP was not specific to liver metastasis, as evidenced by the finding that four patients with elevated AFP levels did not have liver metastases, but rather presented with disease in other areas, including the neck, peritoneum, and chest. This observation suggests that elevated AFP may serve as a marker of tumor de-differentiation, reflecting more aggressive disease biology, rather than merely indicating liver metastasis.

In a cohort of patients with advanced NETs receiving combination chemotherapy with 5-fluorouracil, cisplatin and streptozocin, Turner et al. (2010) examined whether circulating AFP levels were associated with treatment response (16). The study included a total of 82 patients, with 57 patients having available AFP measurements. Elevated AFP levels were defined as greater than 16 ng/mL, which is 1.5 times the upper normal limit. The cohort consisted of 42 males and 40 females, with a median age of 55.4 years (range 22.2–81.6). Tumors were primarily located in the pancreas (60%) and the gastrointestinal tract (40%). Patients with elevated AFP had a higher objective response rate to chemotherapy compared to the response rate in the control group with normal AFP levels (64% versus 25%, respectively, P = 0.020). Despite this, OS did not differ significantly between the two groups, as revealed by multivariable analysis (HR = 1.25, P = 0.58). These findings suggested that elevated AFP was associated with a better response to chemotherapy but did not correlate with improved long-term survival. The study concluded that while AFP may be a predictive biomarker for chemotherapy efficacy, it should not be considered a prognostic marker for OS in advanced NETs.

The relationship between circulating AFP levels and survival outcomes was further examined in a comparative cohort of patients with primary hepatic neuroendocrine neoplasm (PHNENs) and pancreatic neuroendocrine neoplasms (PanNENs) by Li et al. (2019) (17). This retrospective study included 44 patients, comprising 12 cases of PHNENs and 32 cases of PanNENs. AFP levels were assessed using a cut-off value of 20 ng/mL. On univariate analysis, elevated AFP levels were significantly associated with worse OS, with a hazard ratio (HR) of 3.91 (95% CI: 1.259–12.131; P = 0.018). However, after adjustment for relevant clinicopathological variables, including tumor grade and histological type, AFP did not retain independent prognostic significance on multivariable analysis (HR:2.920; P = 0.119). In contrast, serum albumin levels, histological type, and surgical treatment emerged as independent prognostic factors.

The association between circulating AFP levels and tumor grade was explored in a cohort of PanNETs using machine learning (ML)–based predictive models by Zhou et al. (2019) (18). The retrospective analysis included 91 patients with Pan-NETs classified as low grade (G1), intermediate grade (G2), or high grade (G3) according to mitotic count and Ki-67 index. Mean AFP levels increased progressively with tumor grade, from 2.27 ng/mL in G1 tumors to 3.34 ng/mL in G2 and 3.47 ng/mL in G3 tumors, with statistically significant differences observed between G1 and G2 (P = 0.014) and between G1 and G3 tumors (P = 0.035). AFP was incorporated as one of several variables in the ML models developed to predict tumor grade; however, its relative contribution was lower than that of other biochemical markers, including ALT, CA19-9, and CEA.

The potential contribution of circulating AFP to preoperative tumor grading was assessed within multivariable predictive nomograms for GEP-NETs by Li et al. (2021) (19). The study included 1,014 patients overall, among whom 211 were diagnosed with GEP-NETs. Of these, 133 people were put in the training dataset (48 in the G1 group and 85 people in the G2/3 group) and 78 individuals were included in the validation dataset (34 in the G1 group and 44 in the G2/3 group). Serum AFP levels were evaluated alongside other biochemical markers, with a mean AFP level of 12.01 ng/mL in the GEP-NET cohort. On univariate analysis, higher AFP levels were significantly associated with intermediate- and high-grade tumors (G2/G3) compared with G1 tumors (P = 0.046) in the training dataset, but this result was not confirmed in the validation dataset. However, AFP was excluded from the final multivariable nomogram due to its limited incremental predictive value. In contrast, neuron-specific enolase (NSE) emerged as the primary serum biomarker retained in the model, together with clinical variables such as age and sex.

The association between circulating AFP levels and proliferative activity was evaluated in patients with non-metastatic Pan-NENs by Yu et al. (2022) (20). This retrospective study included 142 patients, of whom AFP measurements were available in 126 cases. Patients were stratified into low- and high-proliferation groups using a Ki-67 index threshold of 5%. Mean AFP levels were significantly higher in the high-proliferation group compared with the low-proliferation group (19.20 ng/mL vs 4.02 ng/mL; P = 0.030). Receiver operating characteristic (ROC) analysis demonstrated a modest discriminatory performance of AFP for identifying tumors with higher proliferative activity, with an AUC of 0.626. Although AFP was associated with increased Ki-67 index, its diagnostic accuracy remained limited. In this cohort, CA125 also showed a statistically significant association with proliferative status.

The potential role of circulating AFP as a marker of skeletal dissemination was specifically evaluated in patients with PanNENs by Rosiek et al. (2023) (21). This observational cohort study included 115 patients with PanNENs across disease stages I to IV, the majority of whom had well-differentiated tumors. During follow-up, bone metastases (BMets) were identified in eight patients. Median AFP levels were identical in patients with and without BMets (3 µg/L), with overlapping interquartile ranges (IQRs). Statistical analysis revealed no significant difference in AFP concentrations between the two groups, and ROC analysis confirmed poor discriminatory performance of AFP for the detection of BMets. In contrast, other circulating markers, including CA125 and cytokeratin 18, demonstrated superior diagnostic performance.

The relationship between circulating AFP levels and liver tumor burden (LTB) was examined in patients with well-differentiated GEP-NENs by Jumai et al. (2025) (22). This retrospective study included 200 patients with a median age of 51.4 years, the majority of whom had grade G2 tumors. The AFP/upper limit of normal (ULN) ratio was significantly higher in patients with high LTB compared with those with low liver involvement (median 0.16 vs 0.13; P = 0.004). Despite this association on univariate analysis, AFP was excluded from the final predictive model developed using least absolute shrinkage and selection operator (LASSO) regression. The final model retained Ki-67 index, GGT ratio, LDH ratio, and NSE ratio as predictors of LTB.

Longitudinal changes in circulating AFP over the disease course were analyzed in patients with metastatic Pan-NETs by Musiałkiewicz et al. (2025) (23). This observational study included 41 patients with non-functioning Pan-NETs and liver metastases, with serial AFP measurements collected over a four-year follow-up period. AFP concentrations increased significantly over time across the cohort (P < 0.001). Patients with higher LTB consistently exhibited higher AFP levels compared with those with limited hepatic involvement. In addition, AFP concentrations were significantly higher in patients with grade G2 tumors than in those with G1 tumors (P < 0.001). Rising AFP levels were also associated with radiological disease progression, with patients experiencing progressive disease showing a greater increase in AFP compared with those with stable disease (P < 0.001). No statistically significant differences in AFP levels were observed in relation to age or sex.

The diagnostic and prognostic performance of circulating AFP was evaluated in a single-center cohort of patients with PanNETs by Wan et al. (2025) (24). This retrospective analysis included 43 patients treated over a 20-year period. AFP levels were significantly higher in patients with PanNETs compared with healthy controls, suggesting a potential diagnostic role. ROC analysis showed a modest diagnostic accuracy for AFP, with an AUC of 0.658 at a cut-off value of 15.5 ng/mL. However, AFP elevation was uncommon and did not correlate with tumor grade, disease stage, or clinical aggressiveness. On multivariable analysis, AFP was not identified as an independent prognostic factor; instead, high tumor grade (G3), liver metastases, and advanced AJCC stage emerged as the principal predictors of poor outcome.

## Discussion

**Figure 1:**
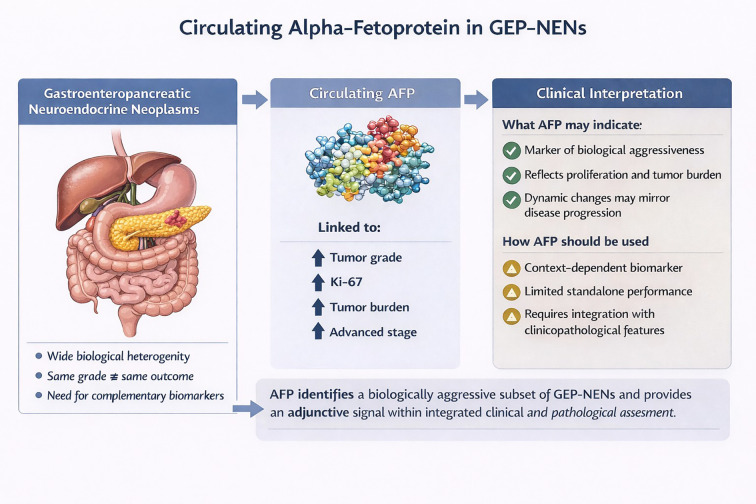
Graphical summary of the potential role of circulating alpha-fetoprotein (AFP) in gastroenteropancreatic neuroendocrine neoplasms (GEP-NENs). Although AFP elevation is uncommon, it is associated with higher tumor grade, increased Ki-67, advanced disease, and greater tumor burden, suggesting a marker of biologically aggressive disease that should be interpreted within an integrated clinicopathological context.

We reviewed ten analytical human studies assessing circulating AFP in GEP-NENs. Across nine retrospective studies and one prospective study, 2,132 patients were included, with AFP measurements available for 1,222. The literature examined heterogeneous endpoints, including associations with tumor grade, metastatic patterns, survival outcomes, and treatment response. A graphical summary of the main findings is depicted in **Figure 1**.

### Diagnostic implications

The diagnostic relevance of circulating AFP in GEP-NENs appears limited to specific subsets of tumors, particularly those with more aggressive biological behavior, such as advanced-stage and high-grade disease. In the largest available cohort, elevated AFP levels were observed in fewer than 10% of patients but were strongly associated with aggressive disease biology, including higher tumor grade and increased proliferative activity, rather than serving as a general diagnostic marker (15). Notably, AFP elevation was not restricted to liver involvement, supporting the concept that AFP reflects tumor biology and aggressive behavior rather than metastatic site specificity.

Evidence from subsequent studies further underscores that AFP should be interpreted with caution, especially in heterogeneous patient populations, and its role remains context dependent. In chemotherapy-treated patients with biologically aggressive GEP-NENs, elevated AFP levels were associated with higher objective response rates but did not translate into improved long-term survival, suggesting a potential predictive, but not prognostic, signal confined to specific clinical settings (16). Similarly, while elevated AFP was associated with poorer outcomes on univariate analysis, this association was not maintained after adjustment for tumor grade and histological features, highlighting the dependency of AFP-related signals on established tumor aggressiveness and pathological determinants (17).

Studies exploring AFP in relation to tumor grade and proliferative activity reported modest correlations, with higher AFP levels observed in G2 and G3 Pan-NETs and in tumors with higher Ki-67 indices (18, 20). However, across multivariable and predictive modelling approaches, AFP consistently demonstrated limited discriminative power compared with other biochemical markers, reinforcing its role as an adjunct rather than a primary diagnostic tool.

### Prognostic implications

Across the available literature, the prognostic relevance of circulating AFP in GEP-NENs appears context-dependent, primarily linked to features of tumor aggressiveness such as advanced stage, high grade, and high proliferative activity, rather than serving as an independent prognostic factor. Early evidence from a large retrospective cohort suggested that elevated AFP levels identify patients with markedly worse OS, particularly in advanced-stage and high-grade disease (15). In that study, patients with elevated AFP experienced substantially shorter survival compared with those with normal AFP levels, supporting a potential role for AFP as a marker of aggressive tumor biology and poor prognosis.

However, subsequent studies have consistently shown that the prognostic signal associated with AFP weakens or disappears when established clinicopathological variables are considered. In a single-center cohort of Pan-NETs, AFP levels were higher in patients than in healthy controls but did not independently predict disease progression or OS in multivariable analyses (24). Similarly, higher AFP levels were associated with intermediate- and high-grade tumors, reinforcing the link between AFP and aggressive tumor biology, yet without demonstrating independent prognostic value beyond tumor grade (19).

This pattern is further supported by studies explicitly adjusting for pathological and clinical confounders. In a comparative cohort of hepatic and PanNENs, AFP was associated with worse survival on univariate analysis but lost statistical significance after adjustment for tumor grade and histological subtype, indicating that its prognostic relevance is largely mediated through established markers of disease aggressiveness (17).

Evidence from LTB–focused analyses provide additional insight into this relationship. Although AFP levels were higher in patients with extensive hepatic involvement, AFP was excluded from multivariable predictive models in favor of biomarkers with greater independent prognostic contribution, such as Ki-67 index and markers of hepatic dysfunction (22). Collectively, these findings indicate that AFP primarily reflects tumor burden and proliferative activity and does not function as a robust standalone prognostic biomarker in GEP-NENs. This consistent pattern suggests that the association between elevated AFP and poorer survival is not independent but is instead mediated by established clinicopathological variables such as tumor grade, Ki-67 index, and disease stage. Thus, AFP appears to reflect underlying aggressive disease features rather than providing separate prognostic information.

### Clinical utility and therapeutic guidance

Evidence supporting a predictive role for circulating AFP in guiding treatment decisions in GEP-NENs remains limited and confined to specific subsets of patients with aggressive tumor biology. In a chemotherapy-treated cohort, elevated AFP levels were associated with higher objective response rates to combination regimens including 5-fluorouracil, cisplatin, and streptozotocin, suggesting that AFP may identify a subset of patients with increased treatment sensitivity (16). Importantly, this association did not translate into improved OS, underscoring that AFP should not be interpreted as a surrogate for long-term treatment benefit in all patients.

Beyond this selected setting, the clinical utility of AFP appears restricted. Studies evaluating AFP in relation to disease progression and metastatic patterns indicate that, while AFP dynamics may reflect tumor burden and progression in metastatic Pan-NETs, AFP lacks sufficient specificity and robustness to independently guide therapeutic decisions (21, 23). These observations reinforce the notion that AFP functions primarily as a contextual biomarker, capturing aspects of disease activity rather than providing actionable guidance on its own. Accordingly, AFP measurement may complement clinical, pathological, and imaging data in selected scenarios, but does not support treatment decisions when used in isolation. Its potential utility lies in integration within multidimensional assessment frameworks rather than as a standalone determinant of therapeutic strategy. Translating the biological signals of circulating AFP into clinical practice requires a framework that balances diagnostic innovation with the need to minimize procedural burden. Pending the availability of robust data from specifically designed prospective trials, a pragmatic integration of AFP assessment within existing clinical workflows is warranted for high-risk subsets, particularly G3 NECs and tumors with extensive liver tumor burden (15, 22).

We propose that AFP monitoring be explicitly synchronized with the standard-of-care imaging and biomarker schedules established for the specific neoplasm (5, 6). This strategy facilitates a synchronized, multi-parametric evaluation of disease status without requiring additional maneuvers or modifying the established therapeutic path. By monitoring AFP dynamics in itinere alongside conventional imaging, clinicians can utilize the marker as a contextual biological sensor (8, 23). This approach may allow for the early detection of lineage escape or clonal evolution, phenomena that is frequently associated with rapid progression, while providing a real-world platform for evaluating AFP’s performance within the current oncological framework (15, 23, 24).

### Discrepancies across studies

Marked heterogeneity characterizes the reported associations between circulating AFP and clinical outcomes in GEP-NENs. While early studies suggested a strong link between AFP elevation and adverse prognosis, particularly in advanced-stage disease and aggressive tumor types, subsequent analyses have demonstrated that this association is highly sensitive to adjustment for established clinicopathological variables. For instance, although elevated AFP levels were associated with poorer survival in univariate analyses, this effect was no longer observed after controlling for tumor grade, proliferative activity, and histological features, indicating that AFP-related signals are largely mediated by underlying tumor aggressiveness rather than representing an independent effect (15, 17).

Discrepancies across studies are further compounded by substantial methodological heterogeneity, including differences in patient populations, disease stage distribution, AFP cut-off values, and analytical approaches. This is exemplified by predictive modeling studies in which AFP demonstrated univariate associations with LTB but was excluded from final multivariable models in favor of biomarkers with greater independent predictive contribution (22). Such findings underscore that the apparent prognostic relevance of AFP is context-dependent and sensitive to model specification. Collectively, these discrepancies indicate that AFP should not be interpreted as a universal or standalone biomarker in GEP-NENs. Rather, its clinical relevance appears contingent on integration with established pathological and clinical parameters, reinforcing the need for cautious interpretation and contextualized use.

### Limitations of AFP as a biomarker

The clinical applicability of circulating AFP as a biomarker in GEP-NENs is constrained by both biological and analytical limitations. A key challenge lies in its limited sensitivity and specificity across clinically relevant scenarios, which hampers its utility for diagnosis, disease monitoring, and risk stratification. AFP elevation occurs only in a small subset of patients and is largely confined to biologically aggressive disease, thereby limiting its ability to serve as a broadly applicable biomarker across the full spectrum of GEP-NENs. For instance, AFP failed to discriminate between patients with and without skeletal metastases in PanNETs, highlighting its inability to capture specific metastatic patterns or disease compartments (21). This limitation underscores that AFP does not reliably reflect disease distribution or metastatic spread, particularly in non-aggressive disease contexts.

In addition, AFP levels may be influenced by factors unrelated to tumor burden or progression, including underlying liver function and systemic conditions, which further complicates their interpretation. Such confounding reduces the reliability of AFP as a diagnostic or prognostic marker, particularly in patients with hepatic involvement or comorbid liver disease, where AFP elevation may not accurately mirror neuroendocrine tumor activity.

From an analytical perspective, variability in assay methodologies, thresholds for defining AFP elevation, and timing of measurement across studies further limit comparability and reproducibility of findings. Together, these biological and technical limitations constrain the clinical value of AFP as a standalone diagnostic or prognostic biomarker in GEP-NENs. Instead, AFP should be interpreted cautiously and, when elevated, considered within an integrated clinical, pathological, and radiological framework rather than as an independent indicator of disease behavior.

An overview of the key findings and constraints across diagnostic, prognostic, and clinical domains is provided in **Table 2**.

**Table 2:** Overview of main findings and limitations of circulating AFP in GEP-NEN.s.

Domain	Main findings	Key limitations
Diagnostic relevance	Elevated AFP levels are observed in a minority of patients and are more frequently associated with biologically aggressive tumors, including advanced-stage disease and high-grade tumors.	Limited sensitivity and specificity; AFP is not specific for neuroendocrine tumor diagnosis and does not reliably distinguish metastatic patterns, including liver involvement.
Prognostic associations	Higher AFP levels are associated with poorer outcomes and higher tumor grade in biologically aggressive GEP-NENs, especially on univariate analyses in selected cohorts.	Prognostic associations frequently lose independence after adjustment for tumor grade, histology, and tumor burden, limiting AFP’s value as a standalone prognostic marker.
Clinical implications	In selected chemotherapy-treated populations, elevated AFP levels have been associated with higher objective response rates, suggesting a potential predictive signal for aggressive GEP-NENs.	AFP does not predict long-term survival and lacks sufficient robustness to guide therapeutic decisions when used in isolation; levels may be influenced by non–tumor-related factors.

AFP, Alpha-fetoprotein; GEP-NENs, Gastroenteropancreatic Neuroendocrine Neoplasms.

### Insights from rare AFP-expressing phenotypes: hepatoid differentiation and lineage plasticity

Although case reports and small series were excluded from the formal analytical synthesis to ensure methodological consistency and mitigate reporting bias (13, 14), they offer critical insights into the extreme biological heterogeneity of GEP-NENs. A qualitative appraisal of this literature identifies specific biologically significant subtypes where AFP production is most prominent. These include gastric and pancreatic neuroendocrine neoplasms exhibiting hepatoid differentiation, a rare variant where extrahepatic tumor cells acquire the morphology and functional capacity of hepatocytes (11). Furthermore, AFP expression is frequently reported in mixed neuroendocrine-non-neuroendocrine neoplasms (MiNENs) with a hepatoid component, where the biomarker reflects a complex interplay between divergent cellular lineages (2).

From a mechanistic perspective, these rare phenotypes exemplify oncofetal reprogramming and lineage infidelity (11). The aberrant production of AFP in these contexts indicates that the tumor cell has hijacked primitive hepatocellular developmental programs, escaping its neuroendocrine commitment to adopt a highly plastic, stem-like state (11). While the exclusion of such cases from the quantitative analysis prevents the overestimation of AFP’s diagnostic sensitivity, their qualitative inclusion in this discussion is essential to understand the biological spectrum of dedifferentiation. These phenotypes suggest that AFP elevation—even in the absence of liver metastases—serves as a contextual sensor of cellular plasticity, identifying GEP-NEN subsets with a unique molecular trajectory and an inherently aggressive clinical course (15).

### Future research directions

Future studies aimed at clarifying the clinical role of AFP in aggressive GEP-NENs should adhere to rigorous methodological and reporting standards, such as those outlined by the REMARK guidelines (25). These guidelines include 20 key items designed to improve the reporting of tumor marker prognostic studies by addressing common shortcomings in study design, analysis, and interpretation. They serve both as an educational resource and a practical reference for researchers, while a condensed version provides quick guidance and facilitates translation into other languages, supporting the global dissemination of best reporting practices.

Adherence to such rigorous standards is particularly important given that the predominantly retrospective nature of the current evidence highlights the need for prospectively designed, multicenter studies focused on biologically aggressive GEP-NENs, with pre-specified hypotheses, clearly defined clinical endpoints, and standardized, blinded assay methodologies. Furthermore, studies should aim to rigorously assess the independent prognostic value of AFP in specific tumor subtypes and incorporate AFP as part of multidimensional clinical and molecular models.

## Conclusion

AFP appears to be a potentially valuable biomarker primarily for identifying specific subsets of aggressive GEP-NENs, characterized by high grade, advanced stage, and increased proliferative activity. While AFP shows limited sensitivity and specificity as a universal diagnostic marker, its role becomes clearer in advanced disease, with potential predictive value in certain chemotherapy regimens. However, it should not be considered a universal prognostic marker, as its relevance is largely dependent on tumor biology. Future research should focus on prospective, multicenter studies to validate the use of AFP in multidimensional clinical-pathological models, aiming for more precise patient stratification and optimized therapeutic decision-making in the field of GEP-NENs.
